# Molecular and Morphological Identification of *Sarocladium* Species Causing Sheath Rot of Rice in Thailand and Their Division into Physiological Races

**DOI:** 10.3390/jof10080535

**Published:** 2024-07-31

**Authors:** Jintana Unartngam, Noppol Kopmoo, Umpawa Pinruan, Chatchai Kosawang, Hans Jørgen Lyngs Jørgensen

**Affiliations:** 1 Department of Plant Pathology, Faculty of Agriculture at Kamphaeng Saen, Kasetsart University, Nakhon Pathom 73140, Thailand; agrjne@ku.ac.th; 2 National Center for Genetic Engineering and Biotechnology (BIOTEC), National Science and Technology Development Agency (NSTDA), Khlong Luang, Pathum Thani 12120, Thailand; noppol.kob@biotec.or.th (N.K.); umpawa.pin@biotec.or.th (U.P.); 3 Department of Geosciences and Natural Resource Management, University of Copenhagen, Rolighedsvej 23, 1958 Frederiksberg C, Denmark; 4 Department of Plant and Environmental Sciences and Copenhagen Plant Science Centre, University of Copenhagen, Thorvaldsensvej 40, 1871 Frederiksberg C, Denmark

**Keywords:** *Sarocladium oryzae*, *Sarocladium attenuatum*, sheath rot, dirty panicle, physiological races

## Abstract

Sheath rot and dirty panicle are some of the major diseases of rice in Thailand. The diseases are traditionally considered to be caused by the pathogen *Sarocladium oryzae* and damage and lower both the quantity and quality of rice grain. In this study, 32 fungal isolates collected from the central and northeastern regions of Thailand were analysed phylogenetically using three molecular markers (ITS, D1/D2 of 28S rDNA and *ACT*) and physiological races were determined on 10 differential rice cultivars. We found that *S. oryzae* is not the only causal agent of sheath rot in Thailand, but *S. attenuatum* was also found. Despite having similar morphological features, the phylogenetic analysis recognised 11 of 32 isolates as *S. attenuatum* and the remaining isolates as *S. oryzae*. This is the first report of *S. attenuatum* causing sheath rot of rice in Thailand in addition to *S. oryzae*. Evaluation of physiological races revealed high pathogenic diversity of the two species. Thus, 16 and 11 physiological races were recorded from 21 isolates of *S. oryzae* and 11 isolates of *S. attenuatum*, respectively. These results indicate that both *S. oryzae* and *S. attenuatum* are the causal agents of rice sheath rot and dirty panicle in Thailand and that they are pathologically diverse.

## 1. Introduction

Rice is attacked by many diseases, with some of the most severe in the panicle stage being sheath rot caused by *Sarocladium oryzae* [1] and dirty panicle caused by a complex of fungi, with *Sarocladium oryzae* being considered the most important causal agent [2,3]. The diseases have been reported in rice growing areas worldwide and are increasing in importance, also in Thailand [3,4,5,6]. The diseases cause significant decreases in the quantity and quality of rice, leading to yield losses up to 85% in susceptible rice cultivars [3,7,8,9]. Symptoms of sheath rot disease include oblong or irregular brown to grey lesions on the leaf sheath near the panicle, which can sometimes prevent panicle emergence when lesions are coalesced [10]. Moreover, the disease can result in grain discoloration, chaffiness and a reduced number of spikelets per panicle [6]. Previously, *S. oryzae* and *S. attenuatum* have been considered as the same species owing to phylogenetic analyses of *S. oryzae* (CBS180.74) and *S. attenuatum* (CBS399.73, CBS414.81) having a similarity of 98.4–98.8% when comparing sequences of three regions (D1/D2, ITS and *ACT*). Moreover, the morphological characteristics of these two species are similar. Therefore, *S. attenuatum* was considered as a synonym of *S. oryzae* [11]. Later, three *Sarocladium* species, *S. oryzae*, *S. attenuatum* and *S. sparsum*, were reported to be casual agents of rice sheath rot in Taiwan [12]. Recently, *Sarocladium* species causing rice sheath rot were reclassified using ITS and *ACT* sequences [1].

While the taxonomy of the genus *Sarocladium* is debatable, control of *Sarocladium* spp. can be challenging because they are seed- and soil-borne pathogens and, therefore, difficult to control [6,13]. Previous studies in various rice growing countries, such as Bangladesh, India, Indonesia, Nigeria and Rwanda, showed great genetic variation of *S. oryzae* using different molecular markers [12,14,15,16]. While molecular markers are a good tool for studying the genetic diversity of an organism, they do not reflect the pathogenicity of the studied pathogen. In contrast, a physiological race is a subgroup within a pathogen species that can only infect a specific set of plant species/cultivars [17,18,19]. Physiological races can be determined using different methods [20,21], including differential host testing and molecular markers. Determination of physiological races often relies on plant accessions involving a gene-for-gene relationship between host and pathogen, and therefore, the knowledge can be directly used in developing resistant cultivars and effective disease management strategies.

In this study, isolates of *Sarocladium* species were isolated from different locations in Thailand from rice showing sheath rot and dirty panicle diseases. The isolates were subjected to phylogenetic analysis of multiple molecular markers in combination with pathogenicity testing on differential rice cultivars. These data will be useful for breeders, who can then plan breeding programmes for rice cultivars resistant to *Sarocladium* species.

## 2. Materials and Methods

### 2.1. Disease Survey and Morphological Identification

Surveys and sampling of rice plants with symptoms of sheath rot and dirty panicle were conducted in paddy fields from 16 provinces in the northeast and central regions of Thailand (Table 1). Panicles and leaf sheaths were randomly collected from 10 points per location, and 10 samples were collected from each point, with 10 m apart in a Z-shape. In total, 100 panicle and leaf sheath samples were collected from each location. The samples were stored in paper bags and kept in a refrigerator at 4 °C until pathogen isolation. The samples were surface-sterilised by 10% sodium hypochlorite (*v*/*v*) for 3 min and then rinsed with sterile water. The tissue transplanting method was used for isolating *Sarocladium* species. Thus, small pieces of tissue were carefully cut from the edges of the lesion and placed on Petri dishes containing potato dextrose agar (PDA). Seeds were surface sterilised as described above before placing them on Petri dishes containing PDA. The Petri dishes were incubated in the dark at ambient temperature for 24 h. Hyphal tip isolation was carried out under a stereomicroscope to obtain pure cultures of *Sarocladium* isolates. The isolates were also grown on potato dextrose agar and oatmeal agar (OA) at ambient temperature. Morphological features of the pure isolates were assessed by examining colony characters, conidia and conidiophores under a compound microscope.

### 2.2. DNA Extraction, PCR and Phylogenetic Analysis

A mycelial mat was prepared by growing 1 mL spore suspension of *Sarocladium* spp. in 250 mL potato dextrose broth (PDB) for 12–18 h. The mycelial mat was harvested, rinsed with sterile water, freeze-dried and stored at −20 °C until use. DNA extraction was carried out using a modified method of Zimand et al. [22]. Briefly, the freeze-dried mycelial mat was ground with a mortar and pestle. An amount of 50 mg pulverised mycelium were mixed with an extraction buffer (200 mM Tris-HCl, pH 8.0, 250 mM EDTA and 0.5% SDS, *v*/*w*) and incubated at 65 °C for 30 min followed by phenol and chloroform/isoamyl alcohol (24: 1 *v*/*v*) extraction. The obtained DNA was washed with 70% ethanol and kept at −20 °C until use.

The internal transcribed spacer (ITS) regions of the rDNA, D1/D2 of 28S and the *ACT* gene were amplified using the primer pair ITS1F/ITS4 [23,24], NL-1/NL-4 [25] and ACT1/ACT4r [26], respectively. PCR was set up using Takara Taq DNA Polymerase (Takara Bio Inc., Shiga, Japan) and 5 µL DNA templates on a Labcycler Basic (SensoQuest GmbH, Göttingen, Germany). The PCR reaction started at 95 °C for 3 min followed by 35 cycles of 95 °C for 30 s, 55 °C for 1 min (or 60 °C for ACT1/ACT4r) and 72 °C for 1 min before final extension at 72 °C for 10 min. PCR products were checked by 1% agarose gel electrophoresis before cleaning up using the PCR clean-up kit (Favorgen Biotech. Corp., Ping Tung, Taiwan) following the manufacturer’s instructions. The purified PCR products were subject to Sanger sequencing at SolGent Co., Ltd. (Daejeon, Republic of Korea). A total of 96 sequences of *Sarocladium* were deposited in the NCBI database (Table 1).

Nucleotide sequences of ITS, D1/D2 of 28S and *ACT* of *S. oryzae*, *S. attenuatum*, *S. sparsum* and other *Sarocladium* species were obtained from NCBI. Multiple sequence alignment of the genes was performed using ClustalW in MEGA X [27]. The concatenated alignment was subject to maximum likelihood (ML)-based phylogenetic analysis using RAxML v.8.2.12 [28] via CIPRES [29] and Bayesian inference (BI) with MrBayes v.3.2.7 [30]. The best sequence evolution model was selected using jModelTest v.2.1.10 [31]. The robustness of the nodes on the obtained phylogenetic tree was evaluated with 1000 bootstrap replications for ML. For the BI, four Monte-Carlos Markov chains (two cold chains) were implemented for 5 million generations, sampled every 1000 generations. The first 25% of generations were excluded as burn-in. The nodes were evaluated with the posterior probability following the Bayesian inference. The tree was drawn to scale, with branch lengths measured in the number of substitutions per site. The analysis involved a total of 41 nucleotide sequences. All positions containing gaps and missing data were eliminated (complete deletion option).

### 2.3. Physiological Race Identification

Spore suspensions were prepared by growing *Sarocladium* isolates on PDA plates for 14 days at room temperature. Spores were dislodged using an L-shaped glass rod in distilled water before mycelial debris were filtered out with sterile gauze. The spore suspensions were adjusted with distilled water to 10^5^ spores/mL before use.

A total of 10 differential cultivars of rice: (Chai Nat 1, RD31, IR72, IR62266, KDML105, Puang Soong 41, Dor Sam Deuan, IR29, Nam Sagui 19 and Puang Tia 41) were used for physiological race identification. Rice seeds were soaked in water for 7 days and one seedling was sown and maintained in 15-inch plastic pots containing clay soil from a paddy field. The pots were kept in a greenhouse at ambient temperature. The inoculation was carried out at the booting stage by placing a drop of spore suspension (10^5^ spores/mL) on the flag leaf sheath. After inoculation, the plants were covered by plastic bags to secure 100% humidity for 48 h. Lesion coverage was measured on flag leaf sheaths at 3, 5, 7, 10, 14 and 21 days after inoculation. Disease severity was evaluated using a six-level scoring system [10] as follows:
**Grade****Symptom**0No symptoms 1Spot lesions < 1% on flag leaf sheath area, and panicle emergence normal2Spot lesions 1–5% on flag leaf sheath area, and panicle emergence normal3Spot lesions 6–25% on flag leaf sheath area, and 75% of the panicle emerged 4Spot lesions 26–50% on flag leaf sheath area, and 50% of the panicle emerged 5Spot lesions 51–100% on flag leaf sheath area, and 25% of the panicle emerged 

Disease index (DI; %) was calculated using the formular:

DI = [(A × 0) + (B × 1) + (C × 2) + (D × 3) + (E × 4) + (F × 5) ÷ (N × 5)] × 100

where A, B, C, D, E and F are number of flag leaf sheaths with disease levels of 0, 1, 2, 3, 4 and 5, respectively, and N is total number of tillers. Rice cultivars with DI between 0 and 25% were considered resistant, whereas those with DI between 26 and 100% were considered susceptible, following the guidelines for surveillance for plant pests in Asia and the Pacific [32].

## 3. Results

### 3.1. Morphology

**Figure 1:**
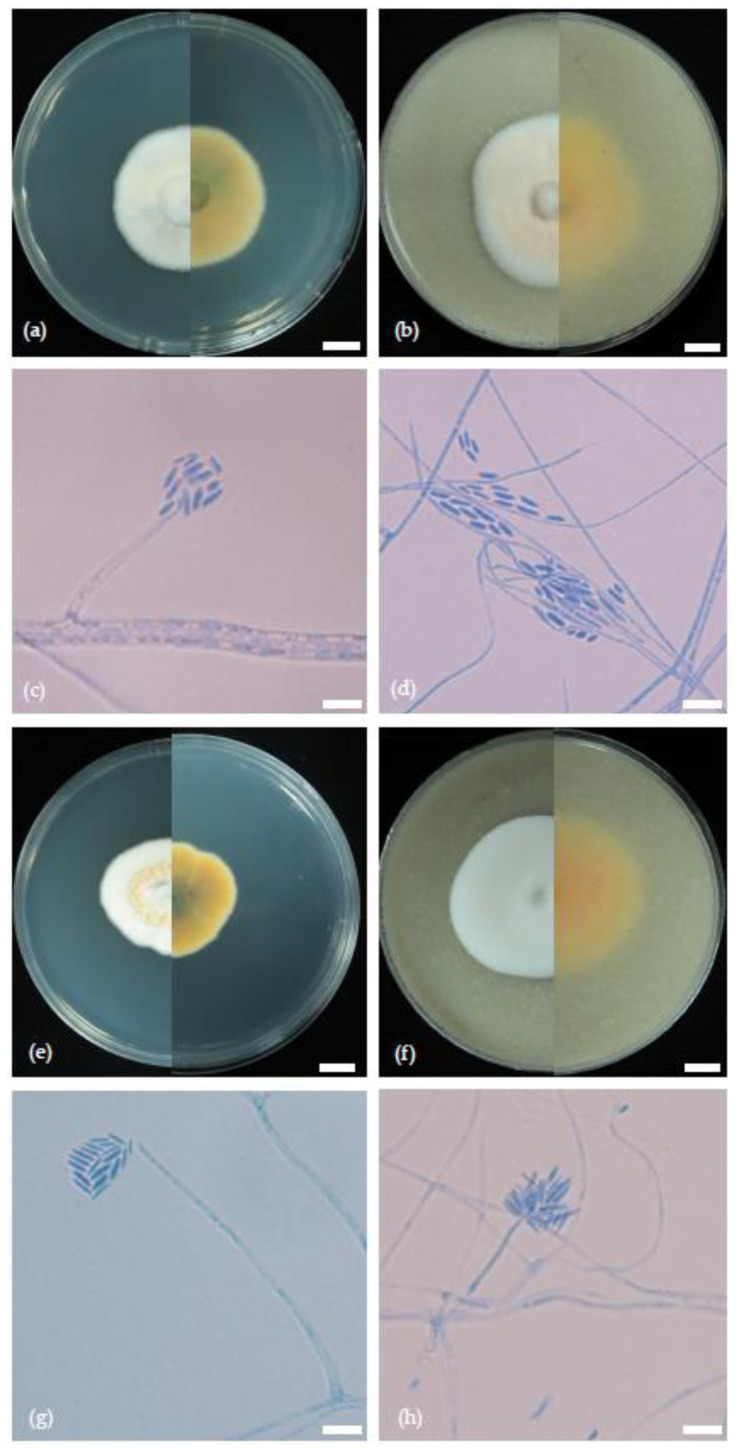
Morphological characteristics of *S. oryzae* (**a**–**d**) and *S. attenuatum* (**e**–**h**) isolated from sheath rot and dirty panicle diseases of rice. (**a**,**b**,**e**,**f**): colonies were grown on (**a**) PDA and (**b**) oatmeal agar (left seen from above and right from below); (**c**,**d**,**g**,**h**): simple and branched conidiophores, phialide producing conidia in chains, cylindrical conidia. Scale bars (**a**,**b**,**e**,**f**) = 1 cm; (**c**,**d**,**g**,**h**) = 10 μm.

The survey of 186 paddy fields from 16 provinces across the country yielded a total of 257 isolates of *Sarocladium* spp. Disease symptoms from the fields are shown in Supplementary Figure S1. Of the 257 isolates, we selected 32 isolates for morphological examination and identified the features of *S. oryzae* and *S. attenuatum*. The following features were observed among isolates identified as *S. oryzae* in this study: vegetative hyphae were septate, hyaline, smooth- and thin-walled and 1–2 µm wide (Figure 1). Conidiophores arising from the mycelium were up to 3 µm in diameter, macronematous, mononematous, erect, single or branched, 15–25 µm long, 1.5–2 (–3) µm wide and hyaline. Phialides were straight or slightly flexuous, subulate, flask-shaped, elongate, narrow towards the apex, 10–20 (–35) µm long, 1.5–2 (–2.5) µm wide at the base, thin- and smooth-walled and lacking any distinct collarette. Conidia were unicellular, cylindrical with rounded ends, sometimes becoming slightly curved, 4.0–6.5 µm (x = 5.2 µm, n = 30) × 1.3–1.8 µm (x = 1.5 µm, n = 30), hyaline to subhyaline, thin- and smooth-walled and arranged in slimy heads. Chlamydospores and sexual morph not observed. Colonies on OA at room temperature (25–28 °C) attained 40–45 mm in 15 days and had a cottony, powdery appearance. Mycelial colour varied from white to light yellow, sometimes turned into pale salmon colour with age (Figure 1). Colonies on PDA at room temperature (25–28 °C) attained 35–38 mm in 15 days. Colony characters varied between white, pinkish white, salmon and orange and were cottony, sometimes radially folded, yellow to pale salmon colour from reverse side, sometimes with a bluish green reverse (Figure 1).

For the isolates identified as *S. attenuatum*, the following morphological features were observed. The vegetative hyphae were septate, hyaline, smooth- and thin-walled and 1–2 µm wide (Figure 1). Conidiophores arising from the mycelium were up to 3 µm in diameter, macronematous, mononematous, erect, single or branched, 15–50 µm long, 1.5–2 µm wide and hyaline. Phialides were straight or slightly flexuous, subulate, flask-shaped, elongate, narrow towards the apex, 10–20 (–35) µm long, 1.5–2 (–2.5) µm wide at the base, thin- and smooth-walled and lacking any distinct collarette. Conidia were unicellular, cylindrical with rounded ends, sometimes becoming slightly curved, 3.0–6.0 µm (x = 4.6 µm, n = 30) × 1.3–1.8 µm (x = 1.3 µm, n = 30), hyaline to subhyaline, thin- and smooth-walled and arranged in slimy heads. Chlamydospores and sexual morph were not observed. Colonies on OA at room temperature (25–28 °C) attained 40–44 mm in 15 days, with cottony, powdery appearance; mycelial colour varied from white to light yellow and sometimes turned into a pale salmon colour with age. 

### 3.2. Phylogeny

**Figure 2:**
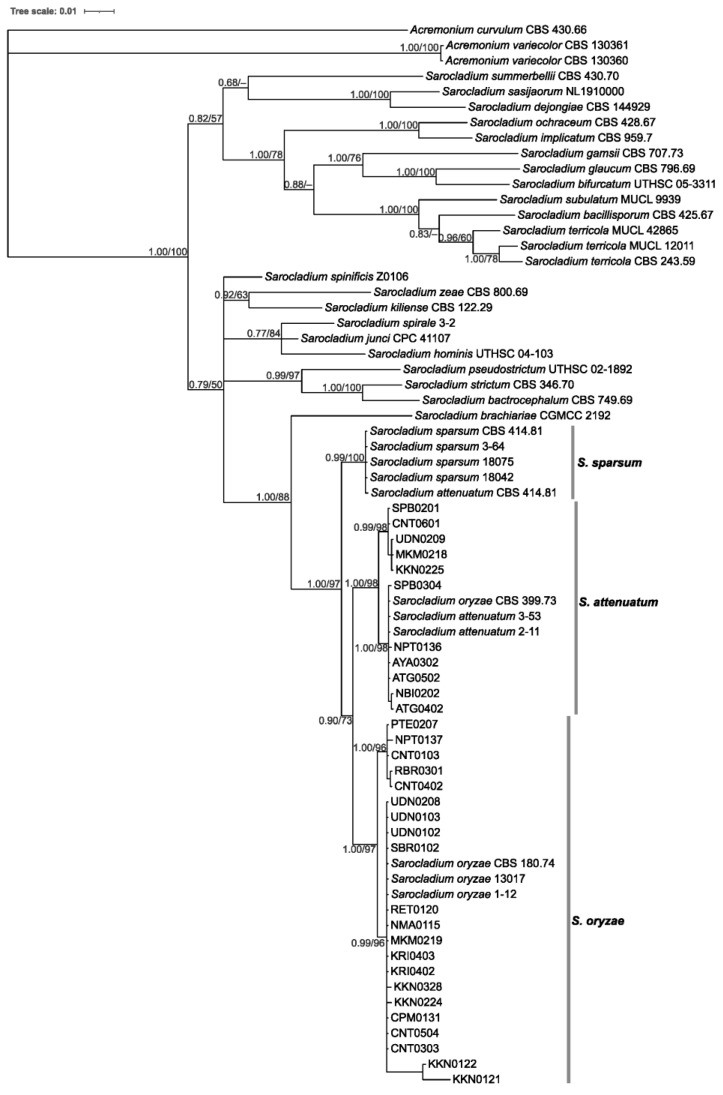
Phylogenetic tree obtained from ITS rDNA, D1/D2 of 28S rDNA and *ACT* concatenated data of *S. oryzae*, *S. attenuatum* and other species from the GenBank database using maximum likelihood and Bayesian inference method. Bootstrap support values (1000 replications) above 50% are shown on the branches.

To confirm the morphological identification, multi-locus phylogenetic analysis of 32 *Sarocladium* isolates (Table 1) and other known *Sarocladium* species was carried out. The analysis made use of 1533 positions in the final dataset and assigned the Thai isolates into two lineages (Figure 2). The first lineage included eleven Thai isolates, one sequence of *S. attenuatum* isotype CBS 399.73, two sequences of *S. attenuatum* 3–53 and two to eleven sequences from the GenBank database with 98% bootstrap value support. The second lineage contained 21 Thai isolates and 3 sequences of *S. oryzae* (ex-epitype culture CBS180.74, 13,017 and 1–12) obtained from GenBank, supported by 97% bootstrap value. *Sarocladium oryzae* CBS180.74 was isolated from rice showing sheath rot. The phylogenetic analysis confirmed the results of the morphological analysis, indicating that sheath rot and dirty panicle of rice in Thailand can be caused by *S. oryzae* and *S. attenuatum*.

### 3.3. Physiological Race Identification

**Figure 3:**
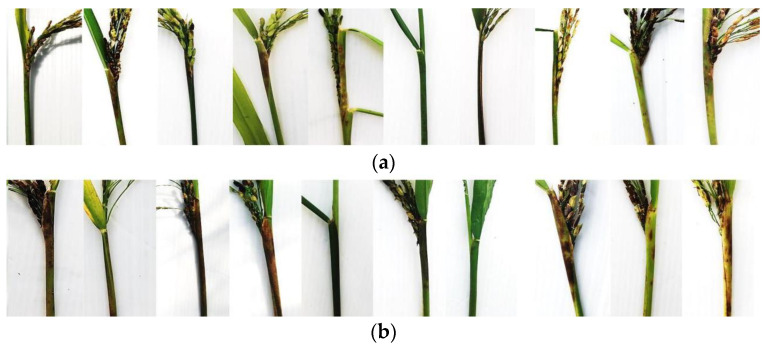
Dirty panicle and sheath rot on panicles and flag leaf sheaths at 21 days after inoculation with a spore suspension of (**a**) *S. attenuatum* isolate KKN0225 and (**b**) *S. oryzae* isolate KKN0122 on 10 differential rice cultivars.

Pathogenicity of 32 *Sarocladium* isolates was evaluated on 10 rice cultivars for fulfilling Koch’s postulates and for race identification. We found 21 isolates of *S. oryzae* isolated from sheath rot forming 16 races (Table 2, Figure 3). All isolates caused disease in at least one cultivar and multiple races were present in one location. For instance, *Sarocladium oryzae* race 1 (SSSSSRRSSS) was distributed in the central [Nakhon Pathom (NPT0137)], north-eastern [Maha Sarakham (MKM0219) and Roi Et (RET0120)] regions of Thailand. Similarly, *S. oryzae* race 6 (SSSSSSRSRS) was observed in north-eastern [Udon Thani (UDN0103)], western [Kanchanaburi (KRI0402)] and central [Sing Buri (SBR0102)] regions of the country.

A nigher number of physiological races was observed in *S. attenuatum*, since 11 isolates were assigned to 11 races (Table 2, Figure 3). It is interesting that multiple races of *S. attenuatum* could be found in the central rice growing regions. For example, race 8 (SPB0201) and race 11 (SPB0304) were present in Suphan Buri, whereas race 9 (ATG0402) and race 10 (ATG0502) were found in Ang Thong. These results validated that *S. oryzae* and *S. attenuatum* are the causal agents of sheath rot and dirty rice panicle in Thailand. In addition, they also showed that the Thai isolates of *S. oryzae* and *S. attenuatum* were physiologically diverse within and among rice growing regions.

## 4. Discussion

Sheath rot and dirty panicle are two of the most important rice diseases found worldwide [3]. In Thailand, only *S. oryzae* has been reported as a causal agent of the diseases [5,7]. Here, we show for the first time that sheath rot and dirty panicle of rice in Thailand can be caused by two closely related *Sarocladium* species: *S. oryzae* and *S. attenuatum*. Several *Sarocladium* species have recently been reported to be associated with rice, including *S. sparsum* and *S. attenuatum* [1,3,12,14,33], but these other species have not been shown to be causal agents of the diseases in Thailand. Since dominant *Sarocladium* species may differ among locations [1], it is possible that more than the two reported species of *Sarocladium* cause sheath rot and dirty panicle in Thailand. However, in the current study, only two species were identified, although the isolates were sampled from different regions of the country.

The taxonomy of *S. oryzae* and its sister taxa has been debated and revised. Originally, *S. attenuatum* was established and differentiated from *S. oryzae* by its verticillate branching pattern of conidiophores and longer conidial length [34]. The species was later synonymised with *S. oryzae* based on phenology and/or multilocus phylogenetic inferences [11,35,36]. However, recent investigations of a larger collection of rice-associated *Sarocladium* strains from Taiwan using multilocus analysis of the ITS/LSU/ACT regions indicated that *S. oryzae* and *S. attenuatum* are indeed two distinct species and that they differ morphologically [12]. Similar results were obtained from Sub-Saharan Africa, where three *Sarocladium* species—*S. oryzae, S. attenuatum* and *S. sparsum*—were reported to be associated with rice sheath rot disease despite using only *ITS* and *ACT* in the study. Our results do not only ratify the existence of S. *attenuatum* as a rice pathogen, but also highlight the importance of multi-locus or even genome-scale phylogenetic analysis for a more accurate species discovery.

The changing climate has been shown to aggravate effects of abiotic stresses on plants [37,38], which may make them more vulnerable to pests and diseases. With the increased risks from sheath rot in Thailand, the first report of *S. attenuatum* as a rice pathogen made in this study sends a critical signal for rice breeders to develop rice cultivars with resistance to sheath rot and dirty panicle, which so far are only available to a limited extent. This is of utmost value considering challenges in finding genetic resources for resistance breeding. Previously, 80 rice lines were evaluated in the field for two years at two locations in search of resistance sources to the diseases and only three cultivars were found suitable for breeding programmes [39]. Another evaluation of germplasm revealed four rice genotypes suitable as resistance donors from a total of 219 rice genotypes screened [40]. The difficulties in finding a donor can be partly due to high variation in pathogenicity of the pathogens [14,40].

As in other studies [1,14,40], we observed that pathogenicity of *S. oryzae* varied among isolates. The variations can be driven by multiple factors, including spatial variations [14,40], as well as diversity of host genotypes [14]. In this study, *Sarocladium* isolates were collected from multiple rice cultivars and from three major rice growing areas of Thailand. The long history of rice cultivation with mixtures of both commercial and local cultivars across the country may be one of the factors facilitating pathogen variation and may explain variation in morphological features and physiological races in this study. However, to what extent diversity of cultivated rice cultivars has influenced diversity and pathogenicity of the two *Sarocladium* species in Thailand needs to be addressed. Additionally, development of differential rice cultivars containing only single resistance genes is also needed as an essential element to understand resistance mechanisms at the histological and molecular level.

The ability of *S. oryzae* to produce secondary metabolites with functions potentially involved in pathogenicity has been noted. Helvolic acid, cerulenin and SO-toxin are among the main pathogenicity factors deployed by *S. oryzae* [16,41], and the infiltration of cerulenin and helvolic acid into host tissues led to electrolyte leakage proportional to the susceptibility to rice sheath rot [42]. Nonetheless, only the production of helvolic acid, but not cerulenin, was later found to correlate strongly with disease severity *in planta* [14]. Whether or not *S. attenuatum* produces these pathogenicity factors, it would be interesting to examine the potential of helvolic acid as a biomarker for screening cultivars in a breeding programme.

In Thailand, a total of 15 rice diseases have been reported [43], and sheath rot and dirty panicle diseases are among those that are not well understood in terms of outbreaks, fungal pathogenicity and management. With an increased incidence of sheath rot and dirty panicle, effective disease management is needed to avoid spread of the pathogen to broader areas and thus causing larger yield losses. Resistant rice cultivars are an essential tool to combat diseases and our study provides knowledge on the pathogens needed for development of a breeding programme and, hence, successful disease control.

## 5. Conclusions

Sheath rot and dirty panicle diseases caused by *Sarocladium* species in paddy fields were present in all Thai provinces studied. Based on DNA sequences, pathogenicity and morphological characteristics, the diseases were caused by both *S. oryzae* and *S. attenuatum*. Studies of pathogenic variation in *S. oryzae* and *S. attenuatum* revealed that 21 and 11 isolates, respectively, were grouped into 16 and 11 races, respectively, based on sheath rot and dirty panicle symptoms.

## Figures and Tables

**Table 1:** List of *Sarocladium* isolates analysed in this study. NCBI accession numbers for ITS/28S/*ACT* for each isolate are listed in last column.

Isolate Code (TBRC No.)	Symptom	Location	Rice Growing Region	Accession No. ITS/28S/*ACT*
NPT0137 (TBRC18772)	Sheath rot	Nakhon Pathom	Central	LC582677/LC652592/LC652753
KKN0122 (TBRC18773)	Dirty panicle	Khon Kaen	Northeastern	LC582679/LC652594/LC652755
KKN0224 (TBRC18774)	Dirty panicle	Khon Kaen	Northeastern	LC582681/LC652596/LC652757
KKN0328 (TBRC10919)	Dirty panicle	Khon Kaen	Northeastern	LC582683/LC652598/LC652759
UDN0102 (TBRC18775)	Dirty panicle	Udon Thani	Northeastern	LC582685/LC652600/LC652761
UDN0103 (TBRC18776)	Dirty panicle	Udon Thani	Northeastern	LC582686/LC652601/LC652762
UDN0208 (TBRC18777)	Dirty panicle	Udon Thani	Northeastern	LC582688/LC652603/LC652764
NMA0115 (TBRC18772)	Sheath rot	Nakhon Ratchasima	Northeastern	LC582691/LC652606/LC652767
MKM0219 (TBRC18779)	Dirty panicle	Maha Sarakham	Northeastern	LC582693/LC652608/LC652769
CPM0131 (TBRC10575)	Dirty panicle	Chaiyaphum	Northeastern	LC582694/LC652609/LC652770
RET0120 (TBRC10635)	Dirty panicle	Roi Et	Northeastern	LC582715/LC652610/LC652771
KRI0402 (TBRC18781)	Dirty panicle	Kanchanaburi	Western	LC582696/LC652612/LC652773
KRI0403 (TBRC18782)	Sheath rot	Kanchanaburi	Western	LC582697/LC652613/LC652774
CNT0103 (TBRC18783)	Sheath rot	Chai Nat	Central	LC582698/LC652614/LC652775
CNT0303 (TBRC18784)	Dirty panicle	Chai Nat	Central	LC582699/LC652615/LC652776
CNT0504 (TBRC18785)	Sheath rot	Chai Nat	Central	LC582700/LC652616/LC652777
CNT0402 (TBRC18786)	Dirty panicle	Chai Nat	Central	LC582701/LC652617/LC652778
PTE0207 (TBRC18791)	Dirty panicle	Pathum Thani	Central	LC582705/LC652621/LC652782
RBR0301 (TBRC18788)	Sheath rot	Ratchaburi	Western	LC582707/LC652623/LC652784
SBR0102 (TBRC18789)	Dirty panicle	Sing Buri	Central	LC582709/LC652624/LC652785
KKN0121 (TBRC18800)	Dirty panicle	Khon Kaen	Northeastern	LC582680/LC652595/LC652756
NPT0136 (TBRC10636)	Dirty panicle	Nakhon Pathom	Northeastern	LC582676/LC652591/LC652752
KKN0225 (TBRC18792)	Dirty panicle	Khon Kaen	Northeastern	LC582682/LC652597/LC652758
UDN0209 (TBRC10918)	Sheath rot	Udon Thani	Northeastern	LC582689/LC652604/LC652765
MKM0218 (TBRC10574)	Dirty panicle	Maha Sarakham	Northeastern	LC582692/LC652607/LC652768
CNT0601 (TBRC18793)	Dirty panicle	Chai Nat	Central	LC582702/LC652618/LC652779
NBI0202 (TBRC18794)	Sheath rot	Nonthaburi	Central	LC582703/LC652619/LC652780
AYA0302 (TBRC18795)	Dirty panicle	Ayutthaya	Central	LC582706/LC652622/LC652783
SPB0201 (TBRC18796)	Sheath rot	Suphan Buri	Central	LC582710/LC652625/LC652786
ATG0402 (TBRC18797)	Dirty panicle	Ang Thong	Central	LC582713/LC652627/LC652788
ATG0502 (TBRC18798)	Dirty panicle	Ang Thong	Central	LC582714/LC652628/LC652789
SPB0304 (TBRC18790)	Sheath rot	Suphan Buri	Central	LC582711/LC652626/LC652787

**Table 2:** Race identification on differential rice cultivars after inoculation by different isolates of *S. oryzae* and *S. attenuatum*.

Race	Strain	Source ^1^	Rice Cultivar ^2^									
			1	2	3	4	5	6	7	8	9	10
***S. oryzae***												
1	NPT0137, MKM0219, RET0120	SR, DP, DP	S	S	S	S	S	**R**	**R**	S	S	S
2	KKN0122, CNT0504	DP, SR	S	**R**	S	S	**R**	S	**R**	S	S	S
3	KKN0224	DP	S	S	S	S	**R**	S	**R**	S	S	S
4	KKN0328	DP	S	S	S	S	**R**	S	S	S	S	S
5	UDN0102	DP	S	S	S	S	**R**	S	**R**	S	**R**	S
6	UDN0103, KRI0402, SBR0102	DP, DP, DP	S	S	S	S	S	S	**R**	S	**R**	S
7	UDN0208	DP	S	S	**R**	S	S	**R**	S	S	S	S
8	NMA0115	DP	S	**R**	S	S	S	**R**	S	S	S	S
9	CPM0131	DP	S	**R**	S	S	S	**R**	**R**	S	S	S
10	KRI0403	SR	S	**R**	S	S	S	S	**R**	S	**R**	S
11	CNT0103	SR	S	S	**R**	S	S	S	**R**	S	S	S
12	CNT0303	DP	S	**R**	**R**	S	S	S	**R**	**R**	**R**	S
13	CNT0402	DP	S	S	S	S	S	S	**R**	**R**	**R**	S
14	PTE0207	DP	S	**R**	**R**	S	S	S	S	S	S	S
15	RBR0301	SR	S	**R**	S	S	**R**	S	**R**	**R**	S	S
16	KKN0121	DP	S	S	S	S	S	**R**	**R**	S	**R**	**R**
***S. attenuatum***												
1	NPT0136	DP	S	S	S	S	**R**	S	**R**	S	S	S
2	KKN0225	DP	S	S	S	S	S	**R**	**R**	S	S	S
3	UDN0209	SR	S	S	S	S	S	S	S	S	S	S
4	MKM0218	DP	S	S	S	S	S	**R**	S	S	S	S
5	CNT0601	DP	S	**R**	S	S	**R**	S	**R**	**R**	**R**	S
6	AYA0302	DP	S	**R**	R	S	S	S	**R**	S	S	S
7	NBI0202	SR	S	**R**	S	S	**R**	S	S	S	S	S
8	SPB0201	SR	S	**R**	S	S	S	S	**R**	S	**R**	S
9	ATG0402	DP	S	S	S	S	S	S	**R**	S	**R**	S
10	ATG0502	DP	S	S	S	S	**R**	S	**R**	S	**R**	S
11	SPB0304	SR	S	R	S	S	**R**	S	**R**	S	**R**	**R**

^1^ Source of symptoms where the isolates were collected from: DP, dirty panicle; SH, sheath rot. ^2^ 1–10 represent the following rice cultivars: Chai Nat 1, RD31, IR72, IR62266, KDML105, Puang Soong 41, Dor Sam Deuan, IR29, Nam Sagui 19 and Puang Tia 4, in numerical order.

## Data Availability

The original contributions presented in the study are included in the article/Supplementary Materials, further inquiries can be directed to the corresponding authors.

## Supplementary Materials

The following supporting information can be downloaded at: https://www.mdpi.com/article/10.3390/jof10080535/s1, Figure S1: Examples of fields, disease symptoms present in the fields and colony morphology of the isolated *Sarocladium* spp.
