# Supplementary material for: Molecular and Morphological Identification of Sarocladium Species Causing Sheath Rot of Rice in Thailand and Their Division into Physiological Races
Source: J Fungi (Basel). 2024 Jul 31;10(8):535. doi: 10.3390/jof10080535 (PMC11355557; doi:10.3390/jof10080535)
Supplement: Supplementary file 1 [file jof-10-00535-s001.zip › jof-3105459-supplementary.pdf]

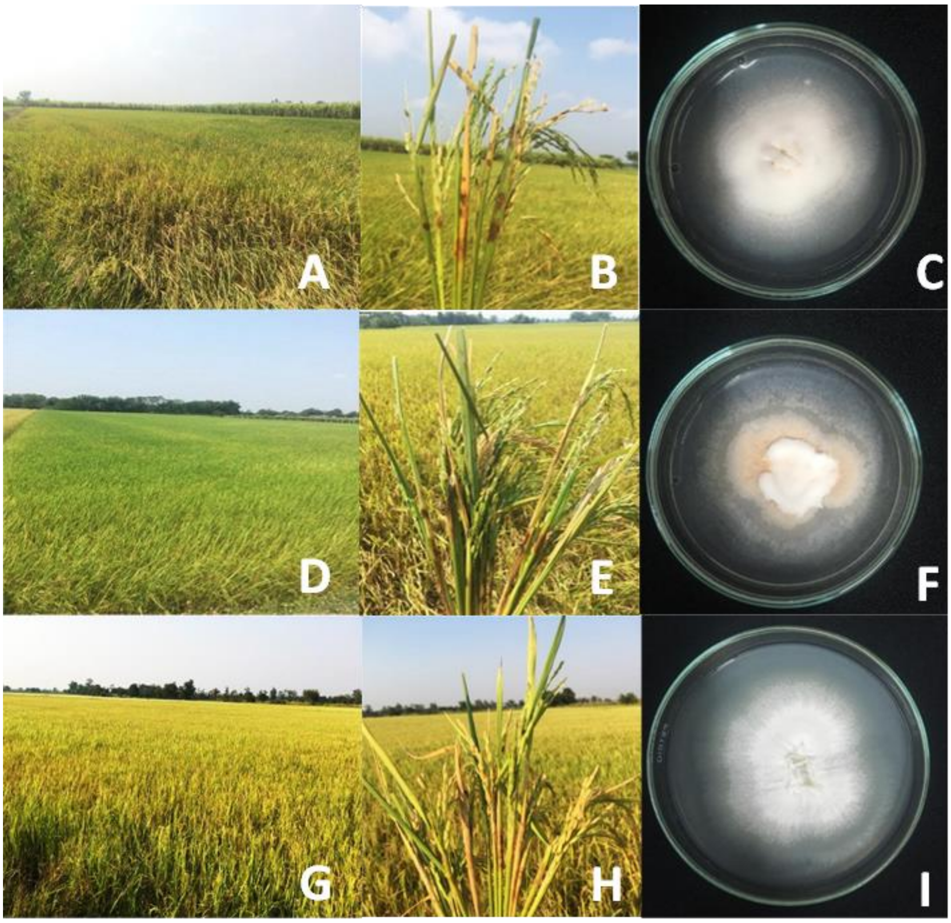

Supplementary Figure S1 Examples of fields, disease symptoms present in the fields and colony morphology of the isolated *Sarocladium* spp. All fields are from one of the collection areas (Sing Buri province) in the central region, but different districts: (A-C) Kai Bang Rajan, (D-F) Bang Rajan and (G-I) Ta Chang.
